# Who Stays, Who Moves on and the Host Population: A Picture of Adolescents’ Perceived Well-Being and Risk Behaviours

**DOI:** 10.3390/ijerph20105902

**Published:** 2023-05-21

**Authors:** Lorena Charrier, Rosanna Irene Comoretto, Michela Bersia, Paola Dalmasso, Emanuele Koumantakis, Alberto Borraccino, Adriana Baban, Paola Berchialla, Patrizia Lemma

**Affiliations:** 1 Department of Public Health and Pediatrics, University of Torino, 10126 Torino, Italy; lorena.charrier@unito.it (L.C.); rosannairene.comoretto@unito.it (R.I.C.); paola.dalmasso@unito.it (P.D.); emanuele.koumantakis@unito.it (E.K.); alberto.borraccino@unito.it (A.B.); patrizia.lemma@unito.it (P.L.); 2 Post Graduate School of Medical Statistics, University of Torino, 10126 Torino, Italy; 3 Department of Psychology, Babes-Bolyai University, 400015 Cluj-Napoca, Romania; adrianababan@psychology.ro; 4 Department of Clinical and Biological Sciences, University of Torino, 10043 Orbassano, Italy; paola.berchialla@unito.it

**Keywords:** adolescents, migrants, host population, natives, country of origin, perceived well-being, risk behaviours

## Abstract

The study aims to evaluate the health profile of first- and second-generation Romanian immigrants living in Italy compared to their adolescent peers in the country of origin (Romania) and the host population (Italian-borns). Analyses were performed on the 2013/2014 Health Behaviour in School-aged Children (HBSC) survey data. Romanian natives showed lower levels of health complaints and higher life satisfaction than Romanian migrants, who were similar to the host population, especially the second-generation ones. A comparable prevalence of being bullied was registered among Romanians, both native and immigrant, with significantly lower levels among Italian natives. Bullying others showed the second-generation migrants share a similar prevalence with the host population. The prevalence of liking school a lot was three times higher among the Romanian natives than among their peers living in Italy. Thanks to the HBSC data, this study is the first to examine the health of adolescent migrants from both the perspective of the host country and the population of origin. The results highlight the need for a more nuanced approach to studying immigrant populations, taking into account both the host country’s perspective and the health patterns of the population of origin.

## 1. Introduction

Scientific literature is rich in studies investigating migration’s impact on adolescents’ health and well-being [1]. However, such research has produced mixed results, often from small studies with different designs and without taking into account the social contexts in which adolescents were living [2,3,4,5,6,7,8].

Young adolescents with an immigration background represent a group of special concern, as they rarely choose to move, but may suffer from their families’ experience of unemployment and poverty, discrimination, stress over adaptation to the host society [7,9,10], and, sometimes, separation from their parents [5]. All these factors have been included in the ‘social stress’ framework, according to which critical stressful life events at a young age, such as involuntary migration, could predict higher depressive symptoms, lower perceived well-being, increased smoking and binge drinking during adolescence, sometimes persistent in adulthood [2,11,12,13,14].

Moreover, the immigration experience was proposed to influence not only the health of the first-generation immigrants but also, indirectly, the second generation through the intergenerational transmission of trauma from parental figures to their children [15,16,17].

According to a study analysing data from 10 different countries, immigration has a negative effect on the emotional and behavioural well-being of adolescents. The study found that first- and second-generation immigrant adolescents experience lower life satisfaction, as well as higher levels of physical fighting and bullying compared to their native peers [18]. Similarly, another recent study revealed that adolescents with a migration background have higher levels of life dissatisfaction and psychosomatic complaints than their native peers. First-generation immigrants also expressed higher levels of life dissatisfaction than second-generation immigrants [19].

Ethnic background and socioeconomic status also appear to be good predictors of adolescents’ perceived well-being and social support [20,21,22]. Indeed, Borraccino et al. showed that immigrants from Eastern Europe and non-Western/non-European countries reported lower life satisfaction compared to their Italian peers from the host population. The difference was even more pronounced in the second generation [21].

From a more comprehensive perspective, first- and second-generation immigrant adolescents face the additional challenge of their “acculturation process” [23], in which two cultures have to be conciliated. This process can lead to opposite results: integration, in which complete incorporation of both cultural norms occurs, or marginalization, in which rejection of both the new and original culture occurs [24,25], with relevant implications in terms of psychological well–being and risk behaviours [26,27]. In light of these conceptualizations, some authors have argued that immigrants should be studied not only from the perspective of the health profile of the host population but also from the health patterns of the population of origin to assess their health properly [2].

The phenomenon of Romanian migration to Italy and the participation of both countries (Romania and Italy) in the Health Behaviour in School-aged Children (HBSC) survey represented an opportunity to investigate the health of immigrant adolescents from the double perspectives suggested above. Romanian immigration to Italy began in the early 1990s, after the overthrow of Ceausescu’s regime in 1989, and according to Istat (Italian National Statistical Institute) data, immigrants from Romania have become the largest ethnic community, with more than 1 million Romanians living in Italy out of about 5 million immigrants [28,29]. Furthermore, Romanians rank first among parents of children born in Italy with the mother or both parents being foreign (about 18% and 22%, respectively) [29]. Nevertheless, a paucity of studies have explored the health of the specific Romanian immigrant community of adolescents in Italy [30,31,32,33]. Therefore, the HBSC data can allow us to cover this gap and analyse perceived well-being and health, going beyond the scope of similar studies comparing the health profile of immigrants living in the host country and natives [20,21,22].

Given these premises, the present study set out to assess the health profile of first- and second-generation Romanian immigrants living in Italy in comparison with their adolescent counterparts in the country of origin (native Romanians), as well as with the health profile and engagement in risk behaviours of the host population (native Italians).

## 2. Materials and Methods

### 2.1. Study Population and Design

The HBSC study is a World Health Organisation (WHO) collaborative, cross-national survey. It runs every four years and provides valuable insight into the well-being, health behaviours, and social context of students aged 11, 13, and 15 (www.hbsc.org, accessed on 16 May 2023). Data are collected through self-administered questionnaires completed anonymously in the classroom. According to the international HBSC protocol, participation is voluntary, and passive or active consent is obtained from school administrators, parents, or adolescents prior to participation and is in line with the regulations in each participating country. Institutional ethical approval is obtained in each country. To ensure consistency of survey instruments, data collection, and processing, each country adheres to the international HBSC survey protocol [32].

We used data from the 2013/2014 HBSC survey, which involved 43 countries across and outside Europe. Specifically, for the aim of the present study, analyses were restricted to Romanian and Italian data, accounting for 40,224 Italian natives’ valid questionnaires, 672 Romanian immigrants living in Italy, and 4815 Romanian natives. A detailed description of the international and Italian HBSC study protocol is available elsewhere [32,33,34].

### 2.2. Variables and Measures

#### 2.2.1. Ethnic Background

Adolescents were asked about their country of birth and that of their parents to determine their ethnic background. According to the HBSC protocol, the ethnic background was defined as the country of birth of the student’s mother. However, if this information was missing or if the mother was born in the survey country, the ethnic origin was defined as the country of birth of the student’s father [32]. The answers reflected into three categories: natives, first- or second-generation immigrants. Specifically, the adolescents involved in the present study were classified as (i) natives if both parents were born in the survey country (Italy or Romania, respectively), regardless of the child’s country of birth; (ii) first-generation immigrants if they and at least one of their parents were born abroad (Romania); and (iii) second-generation immigrants if they were born in the survey country (Italy) with at least one parent born abroad (Romania). No other immigrants included in the original Italian HBSC sample were considered for this study.

#### 2.2.2. Socioeconomic Status (SES)

SES is measured in the HBSC according to the Family Affluence Scale (FAS), recognized as a reliable indicator of family wealth [35]. In the 2013/14 survey, the scale consisted of six items: ownership of a family car, whether adolescents have their own bedroom, number of vacation trips taken in the past year, number of computers owned by the family, ownership of a dishwasher, and number of bathrooms in the home. The score obtained (0–13), and thus the SES, was classified as low (0–6), medium (7–9), and high (>9) [32,35].

#### 2.2.3. Health Complaints (HC)

The subjective HC was assessed through a checklist [36] that recorded the frequency with which participants had experienced the following symptoms in the past six months: stomach ache, headache, backache, feeling low, irritability or bad temper, feeling nervous, difficulty falling asleep, and feeling dizzy. Answers were given on a five-point scale: rarely or never (0), about every month (1), about every week (2), more than once a week (3), and about every day (4). The percentage of students who experience two or more subjective HCs simultaneously, several times a week or daily was calculated.

#### 2.2.4. Life Satisfaction (LS)

The Cantril ladder was used to assess life satisfaction [37]. Participants were asked to indicate their current position on the ladder, with the lowest point representing the worst way of living (score = 0) and the highest point indicating the best possible way of living (score = 10). The results were dichotomized as low (0–5) and high (6–10) life satisfaction [38].

#### 2.2.5. Liking School

Liking school was investigated by asking participants, “How do you feel about school at present?”. The responses, which ranged from “I don’t like it at all” to “I like it a lot”, were dichotomized into “a lot” vs. others.

#### 2.2.6. Smoking and Alcohol Use

Smoking habit was assessed by asking participants how many days they had smoked cigarettes in the past 30 days. Responses ranged from 0 days to every day and were dichotomized into “daily” vs. others. Alcohol consumption was investigated through a question that asked how often, at present, participants drink any alcoholic beverage, such as beer, wine, or spirits. Responses ranged from “never” to “every day” and were dichotomized into “at least weakly” (=weekly or daily) vs. “monthly, rarely or never”. The 2013–2014 HBSC wave was the last to use this question as mandatory, with comparable answers from all participating countries.

#### 2.2.7. Bullying

Bullying behaviours were investigated by asking how often participants had been bullied or had taken part in bullying at school in the past two months. Response options ranged from “never” to “several times a week” and were dichotomized into “none” and “at least once in the past couple of months” [39].

### 2.3. Statistical Analyses

Descriptive data are shown as frequencies and relative percentages (*n* and %). In univariate analysis, the chi-square test was used to compare demographic characteristics and outcomes among the ethnic groups. Logistic regression models were then applied to evaluate the association between the ethnic background and the outcomes of interest after controlling for SES, age, and gender. We checked assumptions on over/underdispersion and heteroscedasticity, potential outliers, and potential confounder variables. All analyses were performed considering the cluster sampling with classes within schools (principal sample unit, PSU).

A statistical significance level of 0.05 was used. Statistical analyses were performed with Stata 16 (StataCorp, College Station, TX, USA: StataCorp LLC).

## 3. Results

The results are based on 40,224 Italian natives, 672 Romanian immigrants (410 and 262 of first- and second-generation, respectively) living in Italy and 4815 Romanian natives.

The distribution of characteristics in the three ethnic groups is shown in Table 1.

The gender distribution was similar when comparing Romanian natives and immigrants living in Italy (*p* = 0.621). We found a significantly higher prevalence of young adolescents—the 11-year-old category—among Romanian immigrants than among Italian and Romanian natives (*p* < 0.001). Moreover, immigrants of the second generation were younger than their first-generation counterparts (*p* < 0.001). A higher prevalence of the low SES category was detected among Romanian natives than immigrants (*p* < 0.001), which was also more than double compared to Italian natives. No differences in SES were found between the two Romanian immigrant generations (*p* = 0.171).

**Figure 1:**
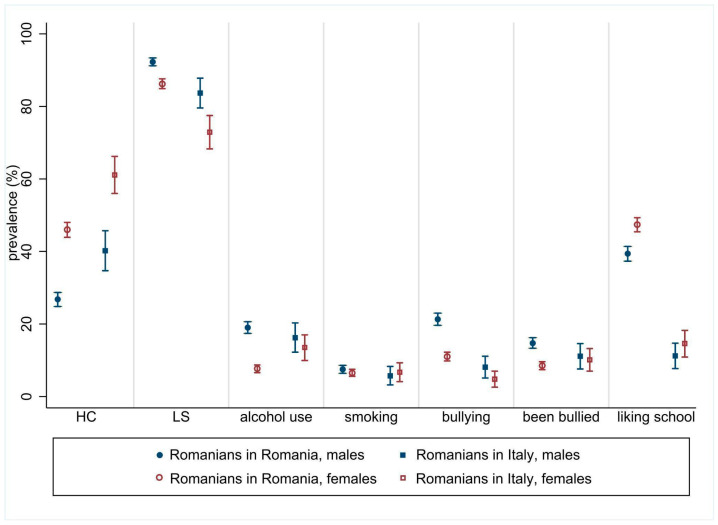
Outcomes (% prevalence and 95% CI) for Romanian natives and Romanian immigrants living in Italy, by gender. Abbreviations: CI, confidence interval; HC, health complaints; LS, life satisfaction.

Figure 1 compares the study outcomes among the Romanian natives and the Romanian immigrants living in Italy, stratified by gender.

Considering the study outcomes stratified by gender, subjective HC had a significantly higher prevalence among females in Romanian natives and immigrants (*p* < 0.001). The prevalence of a high LS was higher among males than females (*p* < 0.001 and *p* = 0.001 for Romanian natives and immigrants, respectively). A significantly higher prevalence of weekly alcohol consumption was detected among Romanian native males than females (19% vs. 7.7%, respectively; *p* < 0.001). Moreover, Romanian native females reported a lower prevalence of frequent alcohol consumption than their immigrant counterparts (*p* < 0.001).

The phenomenon of “bullying others” showed an almost double prevalence among Romanian native males than females (21.3% vs. 11%, respectively) and more than two and a half times higher compared to Romanian immigrant males (8.1%). “Being bullied” turned out to be similar in the two ethnic groups, but a significantly higher prevalence among Romanian native males than females (14.7% vs. 8.5%, respectively; *p* < 0.001) was found. The prevalence of liking school “a lot” was significantly higher among females than males, but the difference was significant only among Romanian natives (47.4% vs. 39.4%, respectively; *p* < 0.001). No differences were found in smoking habits.

The odds ratios (OR) for each study outcome among Romanian immigrants living in Italy compared to Romanian natives are shown in Table 2.

Among the two ethnic groups, subjective HCs (at least two for more than once a week) and at least weekly alcohol consumption were significantly higher among Romanian immigrants than among their native peers. In contrast, the prevalence of high LS, “bullying others,” and liking school “a lot” were significantly lower among Romanian immigrants living in Italy than Romanian natives. No statistically significant differences emerged for daily smoking and “being bullied”.

**Figure 2:**
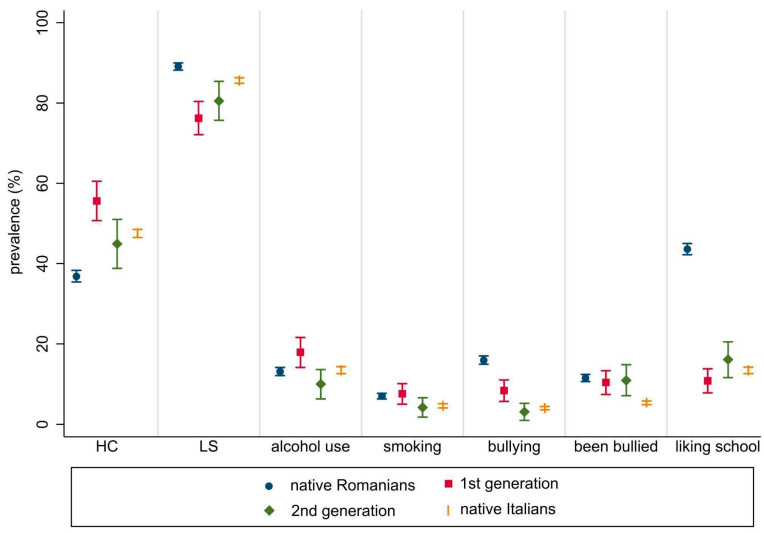
Outcomes for Romanian natives (blue), Romanian immigrants living in Italy (comparing 1st (red) and 2nd (green) generations), and Italian host population (yellow).

Figure 2 shows the prevalence and the associated 95% CI of the study outcomes among Romanian natives, Romanians living in Italy according to the migrant generation, and Italian natives.

Significant differences in subjective HC were found between Romanian immigrants of the first and second generations (55.6% and 44.9%, respectively; *p* = 0.008), with a similar picture for second-generation Romanian immigrants and Italian natives (47.5%). The prevalence of adolescents who reported a high LS level was higher among Romanian natives (89.1%) than among Romanians living in Italy (78%), without differences between the first and second generation (76.2% vs. 80.5%, *p* = 0.191). At least weekly alcohol consumption was higher among Romanian immigrants of the first generation than among their counterparts of the second generation (18% vs. 10%, respectively; *p* = 0.005), while no differences were found when comparing Romanian natives with Romanian immigrants nor Italian natives (13%). Bullying was higher among first-generation than second-generation immigrants (8.4% vs. 3.1%, *p* = 0.007). No differences were detected in being bullied between Romanian natives and Romanians living in Italy, regardless of immigrant generation (11.5% vs. 10.6%, respectively). In comparison, the phenomenon was significantly less frequent among Italian natives (5.4%, *p* < 0.001). If liking school “a lot” was similar among both Romanian immigrant generations and the Italian natives (13%, *p* = 0.119), Romanian natives showed a significantly higher prevalence that reached 44% (*p* < 0.001). No associations with ethnic background were found for the daily smoking outcome.

## 4. Discussion

Studies on the health of immigrants have been mainly based on the adult population and data collected in the host country. In this regard, to provide a more complete overview, some previous literature recommended exploring immigrants’ health patterns not only from the perspective of the host population but also through the lens of the population of origin [2].

Adopting this cross-country approach, the present work used data from the 2013/14 HBSC wave on adolescents’ well-being and engagement in risk behaviour among natives in Italy and Romania and Romanian migrants of the first and second generations living in Italy.

Some results align with other studies comparing immigrants and their peers in the host population. Over half of the second-generation Romanian immigrants were young adolescents (11 years old), whereas first-generation immigrants were more equally distributed among the three age groups. In the low socioeconomic class (low SES), there were more Romanians and fewer Italian natives. In contrast, in the medium and high SES groups, the prevalence of Romanian immigrants was higher among the first-generation group compared to the second generation. These two patterns seemed to indicate the parents’ aim in migrating was to improve the overall economic condition of the family and a desire to remain in the host country [19,20,21].

Furthermore, while findings related to the worse perceived well-being (i.e., subjective health complaints and life satisfaction) among immigrants compared to the host population are well-known patterns in the literature [18,19,21], some interesting results emerged comparing Romanian immigrants and natives. More specifically, Romanian natives presented significantly lower levels of health complaints and higher life satisfaction than Romanian migrants, who resulted in being much more similar in terms of mental outcomes to their peers of the host population, especially the second-generation ones, as already observed in a previous study on immigrants in Italy [30].

The picture that emerged from these findings seems to support the ‘social stress’ framework, according to which critical stressful life events in childhood, such as involuntary immigration or belonging to an ethnic minority group, might predict higher depressive symptoms and negatively affect perceived well-being [11,12,13,14].

Unsurprisingly, very similar profiles were found among natives of both countries and immigrants for smoking and frequent alcohol consumption, likely reflecting the globalizing development of these risk behaviours among adolescents [40,41,42,43,44]. Furthermore, the gender-based comparison of smoking habits showed no significant differences between boys and girls independently from ethnicity, confirming the narrowing of the gender gap previously described in the literature [42,44,45].

Concerning being bullied, a similar prevalence was registered among Romanians, both native and immigrant, with significantly lower levels among Italian natives: this result could suggest that ethnic discrimination in Italy is still a relevant phenomenon [46,47]. On the other side, bullying behaviour (bullying others) showed a decreasing prevalence when comparing Romanian natives and the two generations of Romanian migrants. The second generation shared a similar prevalence of bullying behaviour with the adolescents of the host population. Overall, these patterns could suggest a sociocultural adaptation, where immigrant youths acquire the host country’s culture by learning the appropriate skills needed to operate effectively in a specific social milieu [25]. According to Berry, the acculturation process is a complex path of continuity and change in people’s lives in a new society. This process can be influenced by the welfare policies of host countries as well as individual factors and social resources [9,48,49]. Above all, perceived social support has been found to play a moderating role on the effect of environmental stressors: the lowest probability of reporting low life satisfaction and high health complaints was reported when high support was declared [22,50,51].

Finally, the liking school outcome showed a very clear difference between the Romanian natives and their peers living in Italy, both migrants and hosts, with a prevalence of “liking school a lot” more than three times higher among the Romanian natives. The difference between Italy and Romania was not surprising, as data from the 2013/14 HBSC wave showed Italy ranking at the bottom among the 42 participating countries for liking school, in contrast to Romania, which was among the top 12 countries [42].

Globally, our findings suggested that the burden of migration could play a direct role in worsening perceived well-being among first-generation Romanian immigrants [2,11,12,13,14], especially in the early phase of acculturation. However, results showing similar perceived well-being patterns among second-generation immigrants and Italian natives could enlighten the encouraging outcome of the acculturation process. From this perspective, this research did not detect the effects of the potential generational transmission of migration-related trauma, as previously discussed in the literature [15,16,17,52]. Conversely, the provided evidence contributes to increasing the methodological relevance of considering first- and second-generation immigrants as two separate populations with specific health patterns [18,19,21].

To our knowledge, this study is the first one to specifically examine the health of adolescent Romanian migrants from both the perspective of the host country (Italy) and the population of origin (Romania) on an adequate sample size. This was achieved using data from the Italian and Romanian HBSC 2013/14 survey, which provided the ideal numerosity for such comparisons. In addition, stratification according to immigrant generation was carried out, allowing for a more accurate discussion of the results, given the documented generational differences in health outcomes [30].

Our study has some limitations, too. One limit is that we did not re-test the comparability of the multi-item symptom scale in our sample, as several studies have already validated this type of measure. Another limitation is that the data are from a survey conducted nine years ago and may not reflect current conditions. Nevertheless, in the 2013/2014 school year, a slowdown in the growth of foreign students in Italian schools was observed. This was due to a decline in migration to Italy since 2011 and an increase in foreign-origin children obtaining citizenship and no longer being counted as foreign students in the school system. Furthermore, starting with the 2017/18 HBSC survey, some substantial changes were made to health behaviours questions [53], making them different from previous waves or no longer mandatory. This gives the data used in this study (2013/14 HBSC wave) the ideal characteristics of uniformity and completeness, allowing for a comprehensive examination of the phenomenon.

## 5. Conclusions

In conclusion, this study sheds light on the perceived well-being and risk behaviour engagement among Italian and Romanian natives, and Romanian immigrants of the first and second generations living in Italy.

A negative direct role of immigration-related trauma could be found in the worsening perceived well-being among first-generation Romanian immigrants. On the other hand, the potential generational transmission of migration-related trauma was not detected in our study, as similar levels of well-being were observed among the second-generation immigrants and the host Italian population.

The results highlight the need for a more nuanced approach to studying immigrant populations, taking into account both the host country perspective and the health patterns of the population of origin. Further research simultaneously comparing migrant adolescents with natives of the same ethnicity in other host countries is desirable in order to better discuss potential country-related factors (e.g., school systems and migration policies).

## Figures and Tables

**Table 1:** Demographic characteristics of Italian natives; first-, second-generation, and all Romanian immigrants in Italy; and Romanian natives.

	Italian Natives	Romanian Immigrants			Romanian Natives
		I Gen *	II Gen *	Overall	
	% (*n*)	% (*n*)	% (*n*)	% (*n*)	% (*n*)
Boys	50.1 (20,140)	44.7 (177)	54.4 (138)	46.9 (315)	47.9 (2306)
Girls	49.9 (20,084)	55.3 (232)	45.6 (124)	53.1 (357)	52.1 (2509)
11 years	36.5 (14,689)	33.8 (155)	52.6 (154)	46.1 (310)	30.5 (1463)
13 years	34.3 (13,806)	37.5 (141)	35.4 (74)	32.0 (215)	31.6 (1519)
15 years	29.2 (11,729)	28.7 (113)	12.0 (34)	21.9 (147)	37.9 (1821)
Low SES	26.5 (10,418)	47.5 (174)	38.4 (95)	41.3 (269)	65.6 (2830)
Medium SES	49.8 (19,565)	43.6 (176)	48.3 (119)	45.4 (296)	25.3 (1089)
High SES	23.7 (9291)	8.8 (47)	13.3 (40)	13.3 (87)	9.2 (394)

* Missing data on adolescents’ country of birth and/or their parents. Proportions of both parents being Romanians for first- (I gen) and second-generation (II gen) immigrants were 84.4% and 53.4%, respectively. Abbreviations: SES, socio-economic status.

**Table 2:** ORs (95% CI) for each study outcome among Romanian immigrants living in Italy versus Romanian natives, adjusted for gender, age, and SES.

Outcomes	Romanian Immigrants vs. Romanian Natives OR (95% CI)
Health complaints (2 or more, several times a week or daily)	2.08 (1.74; 2.49)
Life satisfaction ≥ 6	0.34 (0.27; 0.43)
At least weekly alcohol use	1.42 (1.11; 1.83)
Daily smoking	1.18 (0.82; 1.69)
Bullying others	0.31 (0.22; 0.44)
Being bullied	0.87 (0.66; 1.14)
Liking school a lot	0.15 (0.12; 0.19)

Abbreviations: OR, odds ratio; CI, confidence interval.

## Data Availability

The HBSC International Coordinating Centre is based at the University of Glasgow, UK. Data from the HBSC study can be obtained from the HBSC Data Management Centre in accordance with the HBSC data access policy. Further information on accessing HBSC data is available from: https://www.uib.no/en/hbscdata (accessed on 2 March 2023).
